# FUSE: a profit maximization approach for functional summarization of biological networks

**DOI:** 10.1186/1471-2105-13-S3-S10

**Published:** 2012-03-21

**Authors:** Boon-Siew Seah, Sourav S Bhowmick, C Forbes Dewey, Hanry Yu

**Affiliations:** 1 School of Computer Engineering, Nanyang Technological University, Singapore; 2 Department of Biological Engineering, Massachusetts Institute of Technology, USA; 3 Department of Physiology, National University of Singapore, Singapore

## Abstract

**Background:**

The availability of large-scale curated protein interaction datasets has given rise to the opportunity to investigate higher level organization and modularity within the protein interaction network (PPI) using graph theoretic analysis. Despite the recent progress, systems level analysis of PPIS remains a daunting task as it is challenging to make sense out of the deluge of high-dimensional interaction data. Specifically, techniques that automatically abstract and *summarize *PPIS at multiple resolutions to provide high level views of its functional landscape are still lacking. We present a novel data-driven and generic algorithm called FUSE (**Fu**nctional **S**ummary G**e**nerator) that generates *functional maps *of a PPI at different levels of organization, from broad process-process level interactions to in-depth complex-complex level interactions, through a pro t maximization approach that exploits *Minimum Description Length *(MDL) principle to *maximize information gain *of the summary graph while satisfying the *level of detail constraint*.

**Results:**

We evaluate the performance of FUSE on several real-world PPIS. We also compare FUSE to state-of-the-art graph clustering methods with GO term enrichment by constructing the biological process landscape of the PPIS. Using AD network as our case study, we further demonstrate the ability of FUSE to quickly summarize the network and identify many different processes and complexes that regulate it. Finally, we study the higher-order connectivity of the human PPI.

**Conclusion:**

By simultaneously evaluating interaction and annotation data, FUSE abstracts higher-order interaction maps by reducing the details of the underlying PPI to form a *functional summary graph *of interconnected *functional clusters*. Our results demonstrate its effectiveness and superiority over state-of-the-art graph clustering methods with GO term enrichment.

## Background

With advances in high throughput experimental biology, the number of large scale protein interaction net-works (PPI) have grown rapidly. At the same time, collaborative efforts to annotate proteins and genes using Gene Ontology [1] (GO) annotations has generated detailed attributes that describe these entities. Knowledgebases with GO annotations, such as UniprotKB [2], provide a wealth of annotation data at different levels of specificity. GO provides standardized annotations that describe various attributes of a gene or protein, including localization attributes, molecular function, and the biological processes it participates in. As proteins may involve in multiple roles and functions, GO attributes associated with a protein or a gene can be high-dimensional.

While each individual protein or gene has a unique role in the biological system, many of them form communities to govern higher-order biological processes or functions. Biological networks are believed to be modular and hierarchically organized; one may decompose a PPI into modules or *functional clusters *that interact with one another [3]. Protein complexes, for instance, are made up of tightly connected subunit proteins that appear as dense subgraphs in the PPI. Other functional groups may be structurally less obvious. Examples include signaling pathways, where proteins rarely appear to be structurally cohesive. In spite of their "sparse" structure, proteins comprising them share biologically significant signaling propagation function.

### Motivation

The amount of information contained within large biological networks can often overwhelm researchers, making systems level analysis of PPIS a daunting task. As majority of function annotation and high throughput or curated interaction data are encoded at protein or gene level, higher-order abstraction maps such as complex-complex or process-process functional landscapes, are often unavailable. However, availability of such information is invaluable as it not only allows one to ask questions about the relationships among high-level modules, such as processes and complexes, but also allows one to visualize higher order patterns from a bird's eye perspective.

**Figure 1:**
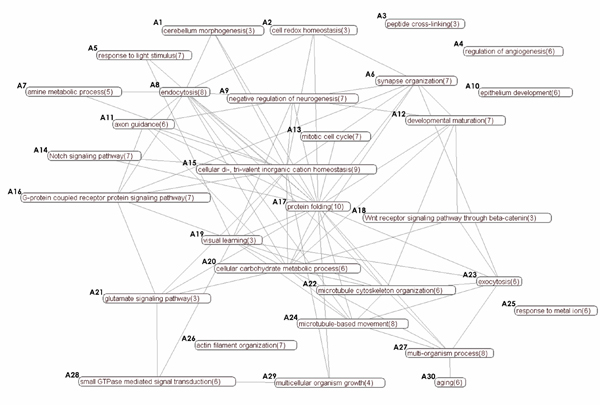
Functional summary (FSG) of the AD network for *k *= 30 (cluster size indicated in brackets).

For instance, consider the Alzheimer's Disease (AD) related PPI in *IntAct *[4]. An AD interaction network can be studied at different levels of organization, from broad-level process-process interactions to in-depth complex-complex interactions. Such maps would reveal higher-level patterns that otherwise would have been invisible. The objective here is not to study a process associated with AD in isolation, but instead focus on the interplay of related processes in tandem to identify the causative mechanisms of AD. For example, one might ask the following questions: How do signaling pathways implicated for AD associate with one another? How do proteins related to transportation play a role in AD, and how are they associated with bioenergetics? A bird's-eye view of the functional landscape of AD network may provide answers to these questions. An example is shown in Figure 1 (detailed in Results Section). Observe that the associations between signaling pathways (*A28*, *A14*, *A18*, *A21*, and *A16 *) are depicted in the summary. It is worth mentioning that it is extremely di cult to answer the aforementioned questions by simply looking at a large PPI containing large number of proteins and interactions as nodes. This problem is further exacerbated by the high-dimensional nature of PPI; each protein may have hundreds of annotation attributes. *It is therefore crucial to have some form of summarization that maps higher-order information of the underlying *PPI. Fortunately, the modular nature of biological networks-either structurally or attribute wise-lends itself to the possibility of building such a summary.

Although tools to abstract high-level and functional information from gene lists have been proven to be key to analyzing high throughput data [5], similar tools that automatically abstract and *summarize *PPIS at multiple resolutions to provide high level views of functional landscape of PPIS are still lacking. At first glance, it may seem that state-of-the-art graph clustering techniques [6-10] can be used for generating high quality summaries of PPIS as these techniques have been successful in identification of novel protein function and protein complexes. Intuitively, a biological network can be decomposed into modules-groups of vertices sharing a common function-that are then collapsed into a representative node to form a summary graph of the underlying network. Depending on the granularity of the decomposition, summaries of various level of detail can be formed. Despite the benefits of graph clustering, these techniques suffer from the following key weaknesses that make them less suitable for building high quality higher order functional summaries of PPIS.

Firstly, several existing graph clustering approaches [6-8,11] overwhelmingly emphasize structure cohesiveness over attribute coherence. In practical applications of PPI summarization, however, attribute coherence is key to forming meaningful, interpretable modules. In PPI, groups of proteins (vertices) that share a common vertex property can form a meaningful cluster that represents a particular biological function. Otherwise, clusters with inconsistent vertex properties, even if structurally well-connected, may not simply summarize into one functionally interpretable cluster. Secondly, majority of existing graph clustering techniques form non-overlapping partitions [6,8,11]. Consequently, they cannot be used to generate high-quality summary because "interactors" in biological processes and pathways are likely to overlap [12]. Thirdly, these techniques typically focus on identifying dense subgraphs from a graph. However, higher-level clusters in PPIS are not always structurally dense. Proteins in signaling pathways, for instance, are structurally loose, but share important functions. Such groups of proteins often have significant biological implications despite their loose structure, and should be present in any summary of the underlying network. Finally, because the annotations that describe proteins and their functions are high-dimensional, finding the right choice of attribute coherent groupings is combinatorial and non-trivial. The reader may refer to [13] for examples related to these limitations.

### Overview

**Figure 2:**
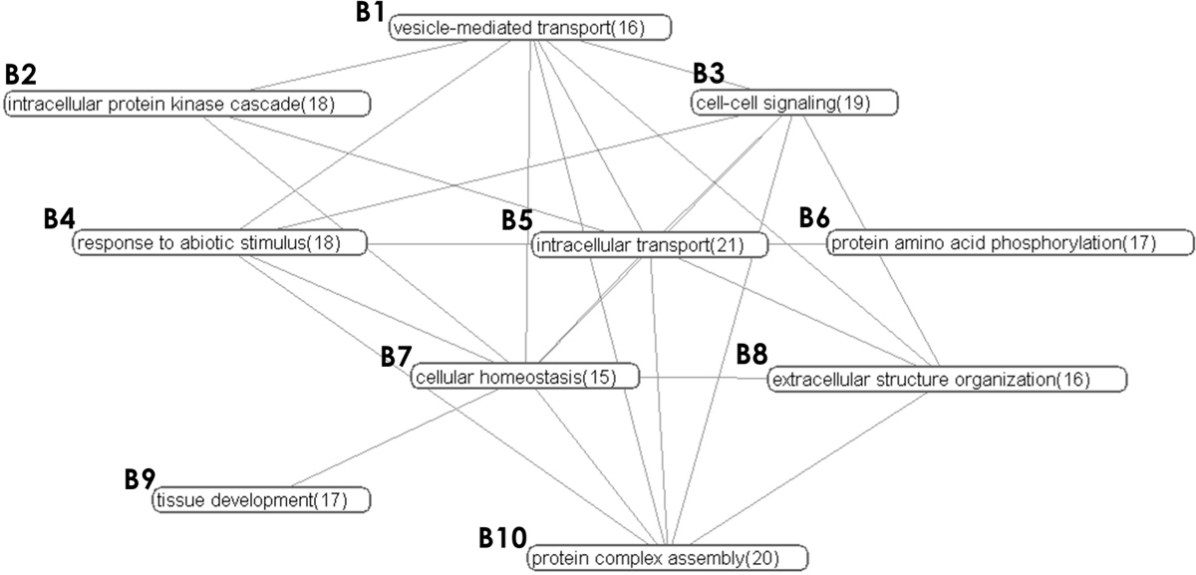
Functional summary (FSG) of the AD network for *k *= 10 (cluster size indicated in brackets).

**Figure 3:**
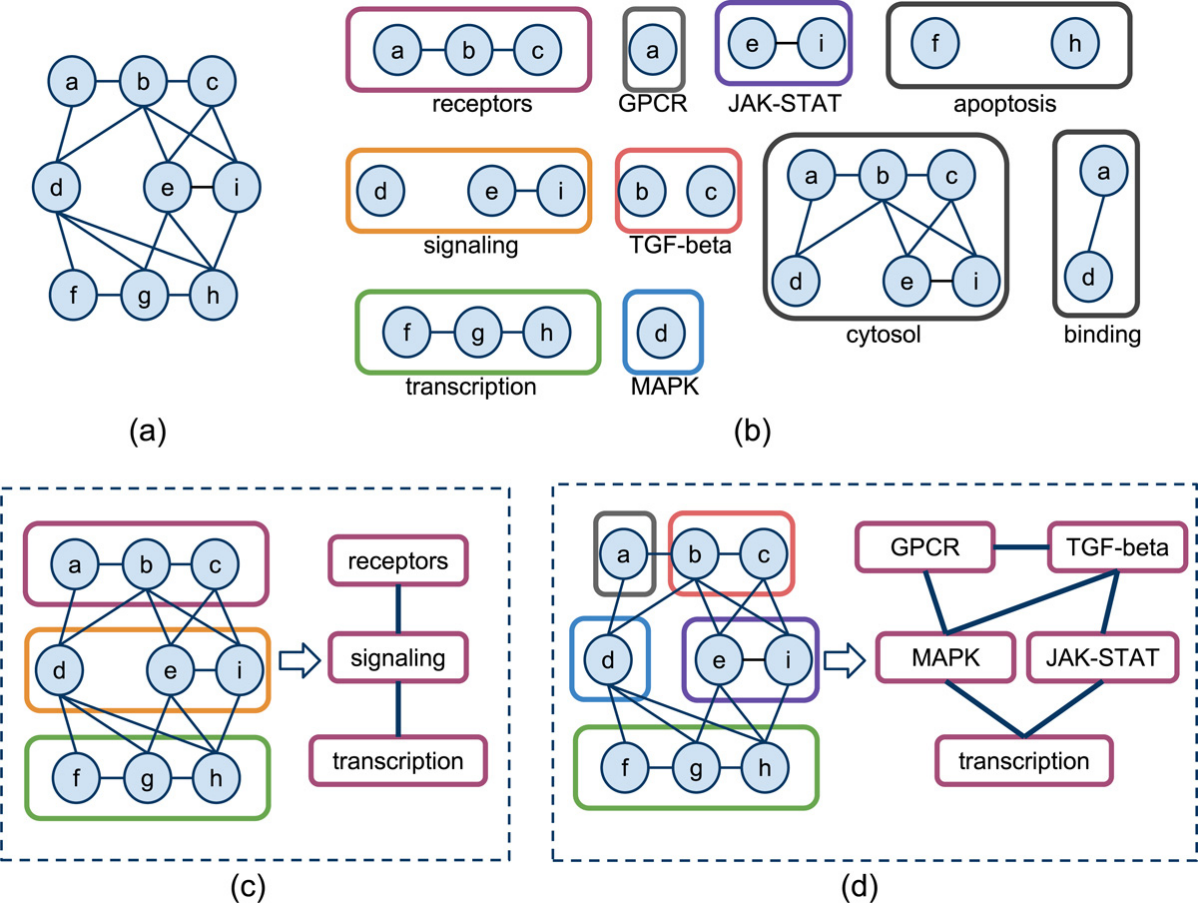
**Illustration of FUSE algorithm.** (a) A toy example of PPI network. (b) A set of *functional clusters *of the network in (a). (c) Suppose a 3-node summary is required (*k *= 3). FUSE explores the functional clusters of the PPI network to identify the 3-node functional summary that best partition and represent the underlying network. This functional summary graph (FSG) depicts the functional landscape of the PPI network in 3 nodes. (d) A 5-node partition (*k *= 5) and its corresponding FSG.

In this paper, we present a novel data-driven algorithm called FUSE (**Fu**nctional **S**ummary G**e**nerator) that addresses the aforementioned challenges (see Methods Section). Given a PPI, it generates a *k*-*node functional summary graph *(FSG) that best represents the higher-order abstraction of the PPI by simultaneously evaluating interaction and annotation data. We argue that a "good" functional summary of a network is not merely a graph of all function-function relationships, but a graph that *reduces *details of the original PPI to form a subset of interconnected *functional clusters*. A *functional cluster *represents a subnetwork of proteins that shares a common function. In particular, the functional summary graph must simultaneously satisfy the following requirements: (a) the summary is at a *specific level *(*k *nodes) of detail, (b) the summary is representative of the original network, and (c) redundancies are minimized. Specifically, FUSE exploits *Minimum Description Length *principle [14] to generate the "best" summary by maximizing *information gain *while satisfying the level of details constraint. Figures 1 and 2 depict a 30-node and a 10-node FUGS of the AD network, respectively, generated by FUSE. Figure 3 depicts examples of functional summaries generated by FUSE.

The goal of FUSE is not only to generate a higher level functional summary that is representative of the underlying PPI, but also to generate a *k*-node functional map whose visual complexity (determined by *k*) permits user analysis. With close to 30000 terms in the Gene Ontology (GO), interaction network of 30000 functional modules will not be a useful summary, as it is just as daunting as the original PPI, if not more. FUSE addresses this challenge by enabling generation of summaries that are small and understandable.

We evaluate the performance of FUSE on several real-world PPIS. We also compare FUSE to state-of-the-art graph clustering methods with GO term enrichment by constructing the biological process landscape of the PPIS. Our experimental results demonstrate that FUSE is highly effective in constructing higher order functional maps with superior accuracy and representativeness compared to these state-of-the-art graph clustering methods. Using AD network as our case study, we further demonstrate the ability of FUSE to quickly summarize the network and identify many different processes and complexes that regulate it. In addition, we analyze the topological features of the functional landscape of human PPI that leads us to the identification of *functional hubs *(clusters of proteins that act as hubs).

### Related work

Functional landscape of an underlying protein interaction network has been explored in [15]. The approach the authors used, however, rely on manual short listing of 229 biological processes for analysis. While this approach makes visualization permissible, it neither scale with the growing number of annotations, nor does it fully utilize the large number of annotations available. Additionally, the processes that are relevant depends on the context of the network.

Graph clustering methods identify functional clusters based on the underlying assumption that the topology of interacting proteins can be mined to identify protein clusters [6-8,11]. Cluster function can then be inferred and annotated by finding enriched annotations within the cluster. While such methods have been proven effective for identification of complexes, they are less suitable for identifying higher level functional clusters, such as biological processes and pathways, where interactors within them are likely to overlap [12,16]. Interactions within a process are also not necessarily cohesive. CFinder [17] locates overlapping communities based on structure of the network, but ignores the wealth of functional knowledge already encoded in GO annotation data. While most graph clustering techniques rely solely on network topology, several recent techniques utilize annotation information when clustering the networks [9,10]. However, these techniques form non-overlapping partitions. Additionally, with the growing amount of annotation data, the attribute space of the nodes in an interaction network is high dimensional as a single protein may be linked to hundreds of annotations. However, these state-of-the-art approaches are not designed for clustering high-dimensional attributes of GO annotated interaction networks. For instance, in [9], a "semantic" distance function is used to measure semantic similarities between nodes with multiple MIPS complex annotations. The *curse of dimensionality *limits the applicability of such an approach on GO annotations [18]. To the best of our knowledge, *no existing method directly addresses our need for generating overlapping clusters from high-dimensional attributed graphs*. Note that existing subspace clustering approaches that allow overlapping subspace clusters typically produce a huge number of clusters that are difficult to interpret [19].

Lastly, the high dependency on interaction topology makes graph clustering ineffective for many context specific networks. Although there are many networks associated with diseases, there are few, if any, with complete interaction knowledge available. The high probability of false positive interactions may also occur. This hampers accurate identification of cohesive clusters.

## Results and discussion

### Experiment settings

We have implemented FUSE in Scala and Java. We now present the experiments conducted to evaluate the performance of FUSE and report some of the results obtained. We used the *coverage *metric to evaluate the fraction of the annotated protein interaction network covered by a summary. A functional summary with high coverage is desirable because it is more representative of the underlying interaction network than a summary with low coverage. Additionally, the *redundancy *metric is the average number of functional clusters each protein belongs to. This is an indicator of the amount of cluster overlap in the summary. Detailed definitions are described in the Methods Section. The PPI datasets employed in this study are shown in Table 1. *Biological Process *(BP), *Molecular Function *(MF), and *Cellular Component *(CC) GO annotations are used. Unless specified otherwise, we set *β *= 0.01, *b *= 3, and *d *= 0 in order to balance *coverage *and *redundancy *of the functional summaries. We assign all edge weights be 1.0. All experiments were run on a 1.66 GHz Intel Core 2 Duo T5450 machine, with 3 GB memory, and a 250 GB SATA disk.

**Table 1:** Summary of datasets used

Dataset	#nodes	#edges	Source
*H. sapiens*	9181	34624	HPRD [37]
*S. cerevisiae*	4768	177299	IntAct [4]
*D. melanogaster*	3114	6472	IntAct
Alzheimer's disease (AD)	177	1038	IntAct

#### Dataset

Currently, there does not exist any gold standard to compare functional summaries of PPIS. Typically, biological graph clustering approaches use MIPS complex annotations [20] as gold standard data for testing cluster quality. These annotations, however, are limited to complexes and not for other functional clusters like pathways. GO annotation data is also used as gold standard for evaluating clustering algorithms. As our approach utilizes attributes of GO, using GO annotations as gold standard evaluation may lead to results biased in favor of FUSE. Instead, we obtained a *different *set of curated attributes as gold standard-the *molecule class *annotations from HPRD-which is distinct from GO attributes. Note that these annotations are only available in the *H. sapiens *dataset. Consequently, we use this dataset for the comparative study. To create a gold standard *reference summary*, we generated a network from subgraphs induced from the HPRD network using nodes grouped by their *molecule class *attribute, signifying the molecular functional groups within the network. Subgraphs from five functional groups corresponding to subgraphs of proteins classified as G protein coupled receptor, Protease inhibitor, RNA binding protein, Cytoskeletal associated protein, and Calcium binding protein are extracted and merged to form the reference summary network (747 nodes, 959 edges). FUSE and state-of-the-art graph clustering methods are then evaluated on this network to determine whether the graph can be partitioned and summarized to reconstruct the gold standard functional groups.

### FUSE vs graph clustering methods

We compare the performance of FUSE with four state-of-the-art graph clustering methods for life sciences applications, namely Markov clustering (MCL) [21], MCODE [6], and NeMo [8]. We also compare FUSE with CSV [11], a recent cohesive subgraph visualization method. Note that in order to obtain higher order modules of a PPI, the current approach is to first use an existing graph clustering method on the network to generate the clusters followed by function assignment. For example, in Krogan *et al*. [21], the global yeast PPI is first clustered using MCL to generate non-overlapping clusters. Then, each cluster is compared against MIPS complex annotations [20] and the complex annotation with the greatest overlap is assigned to represent the cluster.

#### Cluster quality comparison

We first emphasize on the qualities of an ideal summarization. First, the generated clusters have to be representative of the underlying graph, which implies that coverage of the clustering should be sufficiently high. Second, *attribute purity *[22] of the clusterings should correspond to the functional groups that were merged *apriori*. This can be determined through the purity of the molecule class attribute within the proteins in each cluster. Each functional group should also be well-represented. We use *precision*, *recall*, and *F-measure *to quantify these features. For each cluster, we determine the molecule class functional group that best matches the cluster. The *purity *of that cluster is then defined as the proportion of nodes in the cluster that belong to the best matching group. As a functional group may be represented by several smaller clusters, we define *recall *for each functional group as total coverage of the functional group among the clusters that best matches that functional group. Then, the *precision *of a clustering is defined as the average purity among all clusters. The *recall *of a clustering is defined as the average recall among all functional groups. Lastly, the *F-measure *$\left(\frac{2^{*}precision^{*}recall}{precision+recall}\right)$ provides an overall measure of clustering quality.

**Figure 4:**
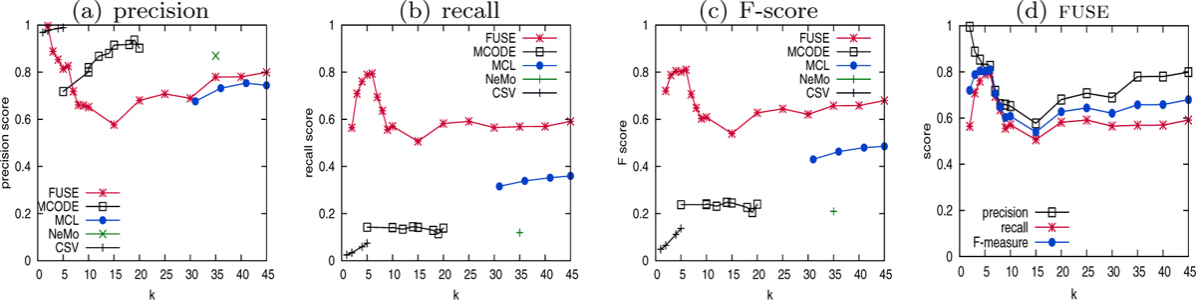
Cluster quality of FUSE vs graph clustering-based approaches.

Figure 4 depicts the results of summarization quality. Where applicable, we adjust relevant parameters to vary the cluster granularity. As NeMO has no parameter to tweak, only a single set of clusters can be obtained. In MCL, CSV, and MCODE, the *inflation*, *η_mseen _cutoff*, and *node score cuto *parameters are adjusted, respectively, to vary the cluster sizes (denoted as *k *in all figures). In FUSE, the parameter *k *directly affects the summary granularity.

Observe that FUSE generates summary with significantly higher F-measure score compared to the graph clustering-based approaches for all values of *k*. In other words, FUSE may generate summaries at multiple levels of complexity while remaining representative of the underlying graph. Observe that, although NeMO, CSV, and MCODE generate clusters with high precision, the recall scores are very low (< 0:2). This is because these two approaches identify highly cohesive subgraphs, which tend to be part of protein complexes. CSV in particular are limited to identification of near-clique structures. Proteins in complexes belong to the same functional groups and hence the high precision. However as mentioned earlier, biological networks are not comprised solely of complexes. Consequently, majority of the underlying network was poorly represented by these approaches due to heavy bias towards complexes. Specifically, most of the clusters match the RNA binding protein class of proteins, leaving other groups barely represented. For instance, the Protease inhibitor subgraph is not well represented because of its inherent loose structure. Although the recall score of MCL is relatively higher as this method is known to perform very well in biological clustering applications, it is still below 0.4. Note that the MCL approach failed to partition the underlying network into five clusters representing the five functional groups. The CSV approach, on the other hand, were not able to generate larger number of partitions.

Notice that these existing approaches indirectly affect the summary complexity whereas FUSE allows direct adjustment of summary size, which explains why summaries at any level of detail can be obtained by the latter. Figure 4(d) shows that FUSE generates summaries at different granularity without greatly affecting the precision and recall of the clusterings. The peak F-measure score of 0.8 is obtained in FUSE at *k *= 5, corresponding to the five gold standard functional groups that comprise the dataset. Observe that the recall and precision scores are equally high. As cluster granularity deviates from the underlying five functional groups, obviously the F-measure score drops.

#### Function representativeness comparison

The accuracy and representativeness of the function assigned to each cluster is key to generating high quality functional maps. Here, we introduce measures that quantify the representativeness of functions assigned to each clusters and compared FUSE to graph clustering methods in this aspect.

To obtain the functional landscape of a PPI, graph clustering methods often assign function to clusters through functional enrichment techniques. To this end, we compute the statistical significance of association of the cluster with every GO term based on the hypergeometric distribution [5]. The term with the best *p-value *is assigned as the *representative function*, denoted by *a_r _*∈ Δ, of the cluster. To evaluate the representativeness of this assigned function, we reuse the precision and recall measures introduced earlier with slight modification. Specifically, the *representative purity *of a cluster *C *is defined as the proportion of nodes in the cluster that are annotated with the representative function, *i.e. $\frac{\left|\left\{v\in C:{▵}_{v}\left[a_{r}\left(v\right)\right]=1\right\}\right|}{\left|\left\{v\in C\right\}\right|}$*. We also define *representative recall *for each functional group as total coverage of the functional group among the clusters that has the functional group assigned as representative function, *i.e. *$\frac{\left|\left\{v\in C:{▵}_{v}\left[a_{r}\left(v\right)\right]=1\right\}\right|}{\left|\left\{v\in V:{▵}_{v}\left[a_{r}\left(v\right)\right]=1\right\}\right|}$. Then, the *precision *of the representative functions is defined as the average representative purity among all clusters, and the *recall *of the representative functions is defined as the average representative recall among all functional groups.

**Figure 5:**
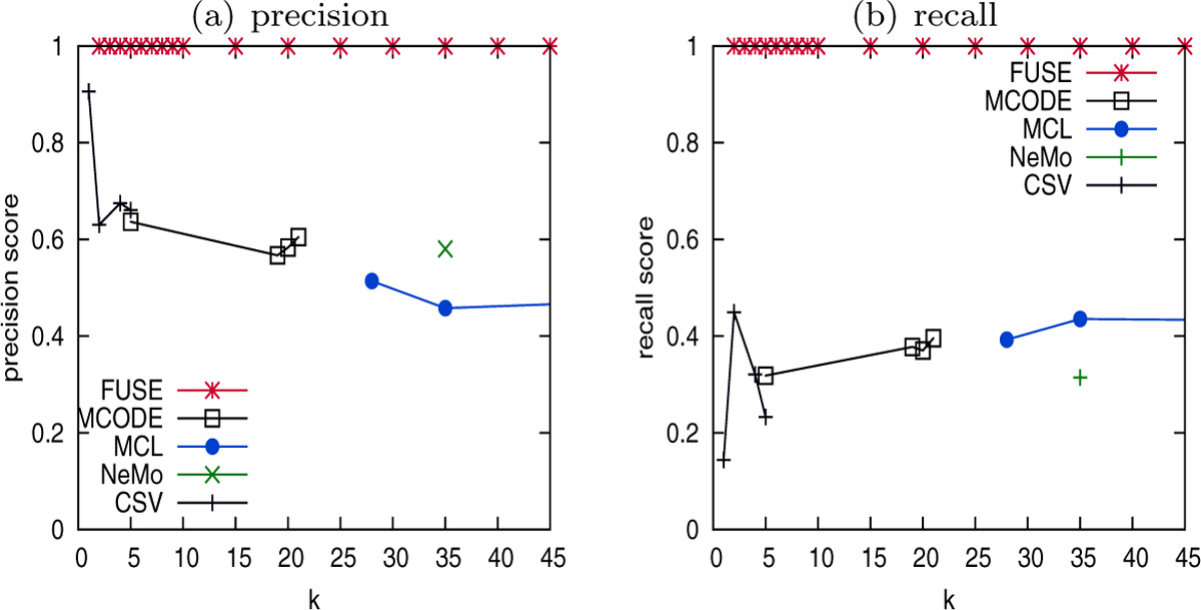
Function representativeness.

Figure 5 depicts the representativeness of the functional summaries by different techniques. As FUSE is designed specifically to generate highly representative maps, each cluster is perfectly representative of the biological function assigned to it. Likewise, each function is well represented by its assigned cluster. In graph clustering methods, however, the clusters do not represent their representative function well, as indicated by the lower precision score. Hence, proteins within the clusters exhibit less functional coherence. The lower recall scores in graph clustering methods imply that only a fraction of nodes annotated with the representative function are included in the cluster. That is, FUSE summaries contain functional clusters that are more representative of the assigned function, and thus provide more meaningful and interpretable higher-order functional maps of the underlying PPI. While clusters without attribute coherence may still reveal novel biological insights, assigning a function to represent such cluster could be misleading.

### Effects of user-defined parameters

#### Effect of parameter *k*

**Figure 6:**
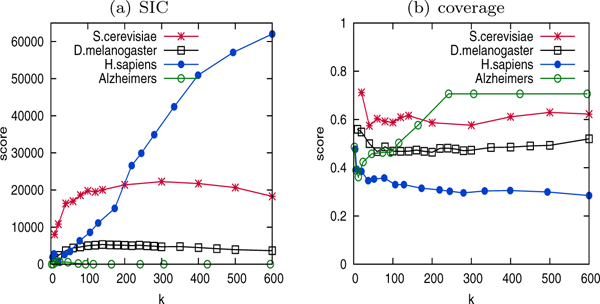
Effect of *k.*

Recall that the user-defined parameter *k *controls the granularity of the summary. Intuitively, as *k *increases the amount of information contained within the summary as well as its complexity increase. Figure 6(a) reports the effect of *k *on the summaries of test datasets. As *k *increases, the *summary information content *(SIC), denoted by *SIC*(Θ), rises rapidly until it saturates to a peak value before tapering off.

(1) $$SIC\left(\Theta \right)=\underset{C\left(u\right)\in S_{⊖}}{\sum }-\psi^{C\left(u\right)}|V\left(u\right)|logp\left(V\left(u\right)\right)$$

where *p*(*V *(*u*)) is the probability that a protein in network is annotated with term *u *or its descendants. Note that summary profit cannot be used for comparing summaries with different *k *values because it does not make any assumption about the information content of a GO term attribute. In contrast, sic measure is summary profit with a twist - small clusters are weighted higher than large clusters. This allows one to compare information content of summaries with different *k *values. Other factors being equal, a summary with many small clusters will contain more information than a single large cluster. The above results imply that *k *is useful up to a certain value, after which increasing *k *only increases summary complexity while providing little information gain.

Figure 6(b) plots the effect of *k *on coverage of the summary. Observe that except for low *k *values, it is relatively stable as *k *varies. In fact, the amount of information a summary can provide is limited by the resolution and completeness of the interaction and annotation data. This could explain why *S. cerevisiae *summaries have consistently higher coverage and information content than *D. melanogaster *summaries. The *H. sapiens *summary contains the largest number of nodes and edges, and even at *k *= 600, information content is still increasing. The AD network reaches a peak of information content at *k *= 20.

#### Effect of parameters *b *and *d*

**Figure 7:**
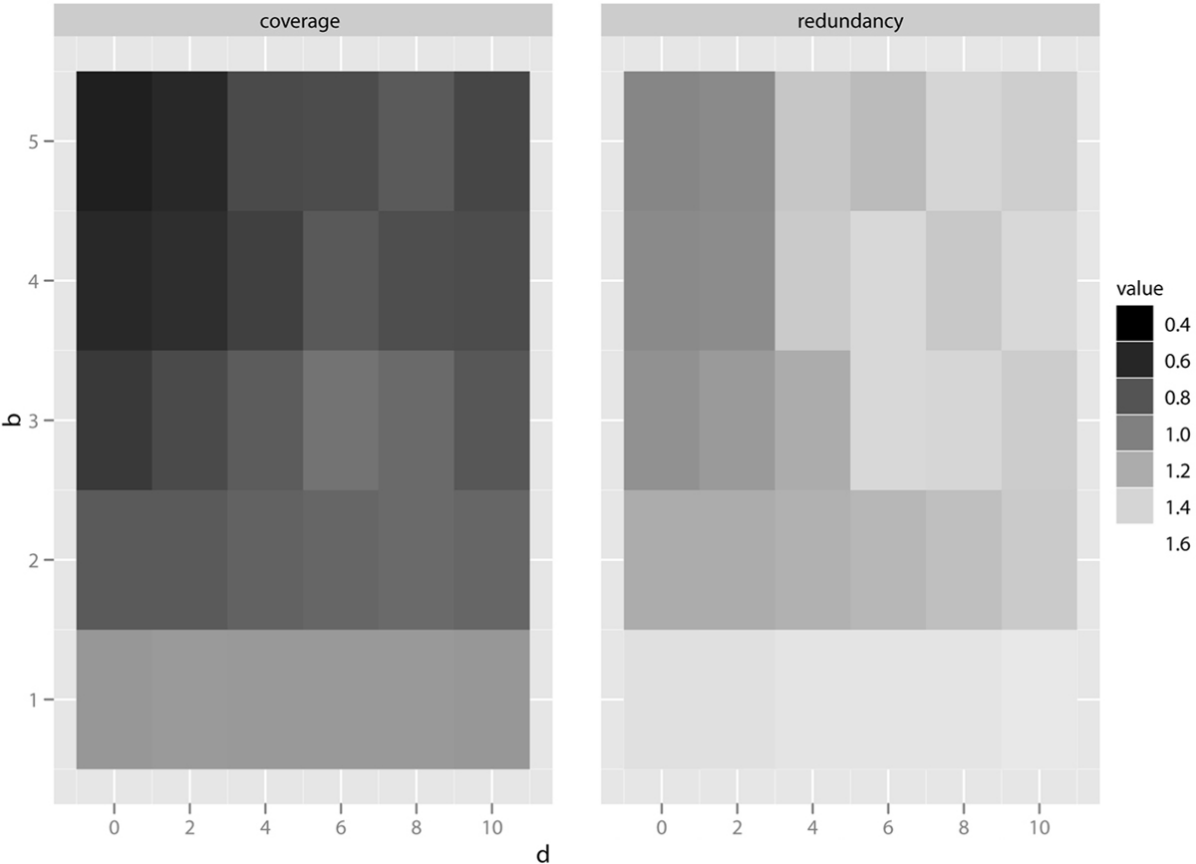
Effect of b and d.

We investigated the effect of user-defined parameters *b *and *d *on summary coverage and redundancy. We use the global *S. cerevisiae *dataset with *k *= 100. Figure 7 shows that increasing *b *or decreasing *d *lowers overall summary redundancy at the expense of lower summary coverage. On the other hand, when *d *is increased or *b *is decreased, both summary redundancy and coverage increases. An intuitive explanation of this phenomenon is that more cluster overlap penalty means fewer combination of clusters to choose from, lowering the likelihood of finding a summary with high coverage. Both parameters allow users to control the coverage and redundancy tradeoff.

### Runtime and scalability

**Figure 8:**
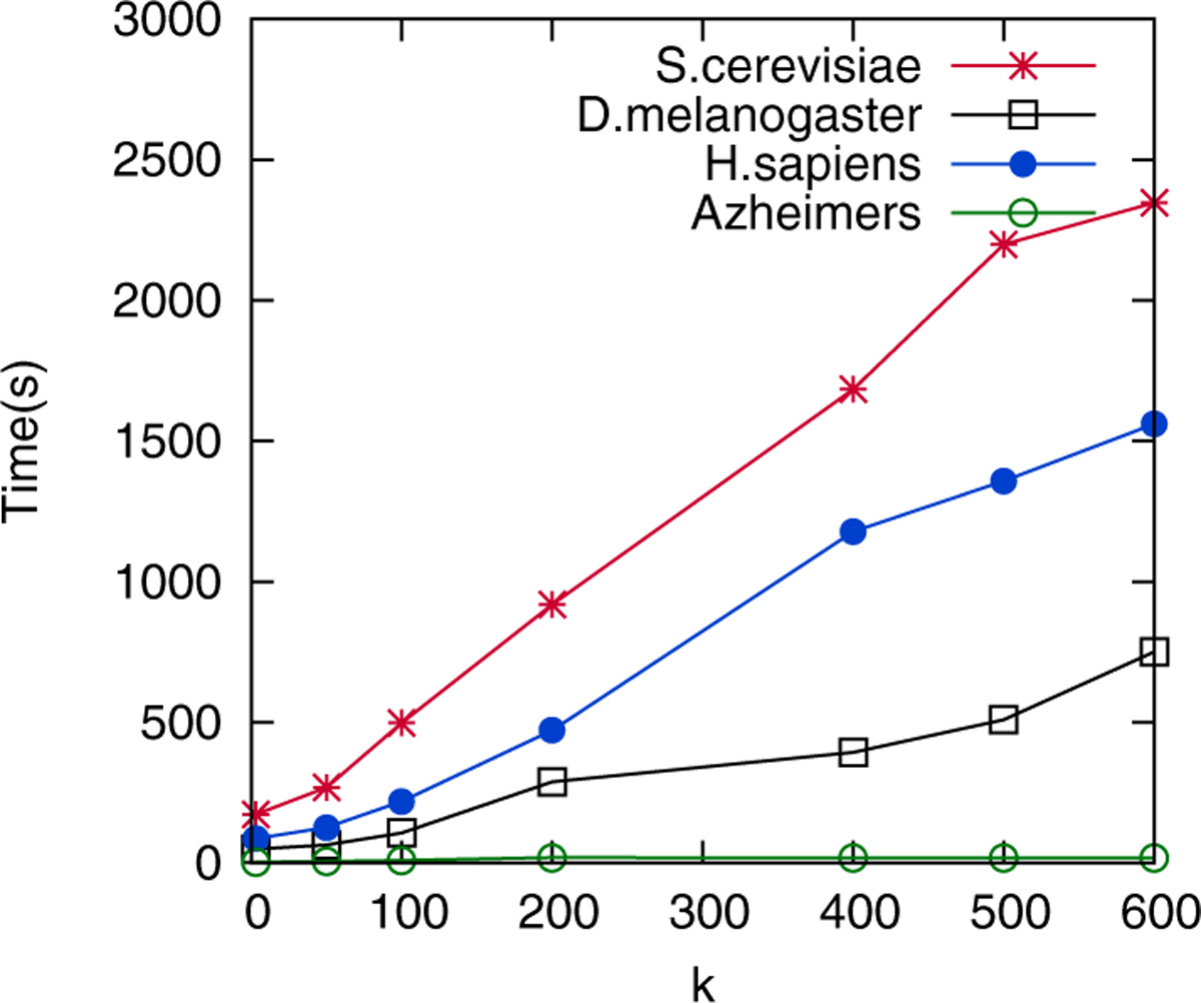
Running times of FUSE (in sec.).

Figure 8 plots the running times of FUSE over the real datasets for generation of summaries ranging from *k *= 3 to *k *= 600. Observe that it increases almost linearly with *k*. Specifically, summarization of the yeast interaction network (the largest available network) completes within 40 minutes for *k *= 600. For practical sizes of *k *= 3 to *k *= 100, a functional summary of a PPI can be generated within few minutes. Disease networks such as AD network can be completed in less than 10 sec.

We now assess the scalability of FUSE with respect to network size and *|S*_Δ_*|*. Note that the latter feature is important as it will continue to grow as more annotation information becomes available. To assess the scalability with respect to network size, we generated synthetic networks of vertex size |*V *| = 100 to |*V *| = 20000. For every term *t*, a vertex has a 2% probability of being annotated with it. The number of terms is *|S*_Δ_*| *= 2769. The *edge density *of the synthetic networks is such that the probability that a pair of vertices interact is 0.0025, resulting in an average of 1 million edges in a network of 20000 vertices. Summary granularity is set to *k *= 50. To measure the effect of *|S*_Δ_*| *on running time, we generated synthetic networks by varying *|S*_Δ_*| *ranging from *|*Δ*| *= 100 to *|*Δ*| *= 10000.

**Figure 9:**
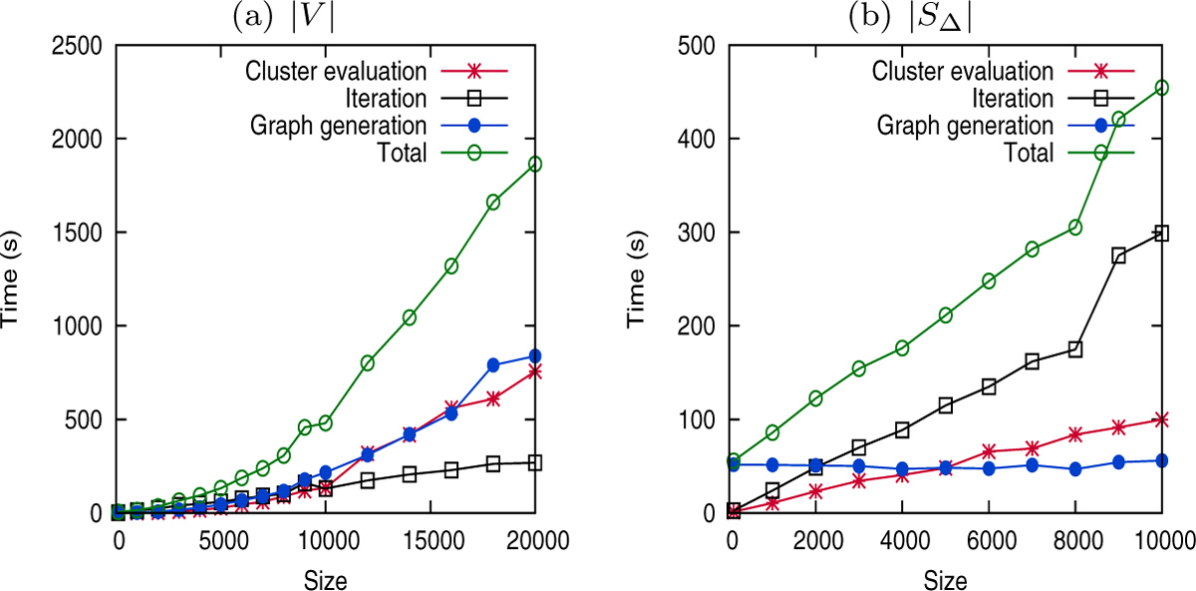
Scalability of FUSE.

Figure 9 depicts the scalability of FUSE with respect to *|V | *and *|S*_Δ_*|*. As the number of vertices increases, the execution time of FUSE increases in a quadratic fashion. In fact, it appears to increase almost linearly for networks with |*V*| < 10000. For larger networks, the *ψ*^*C*(*u*) ^component and the fsg generation component take up the bulk of the execution time. Observe that in Figure 9(b), the fsg generation component takes up bulk of the computation time and is independent of *|S*_Δ_*|*. As *|S*_Δ_| increases, *ψ*^*C*(*u*) ^computation and iterative cluster selection time increases in near linear fashion, demonstrating ability of FUSE to handle high-dimensional annotation data.

### Case study on AD network

In this section, we construct a low and a high resolution functional summaries of the AD network to illustrate the benefits of FUSE in providing a higher level functional view of the underlying PPI. A low resolution summary delineates broad functional overview of the processes related to the disease whereas a high resolution summary provides in-depth functional landscape of the disease, revealing associations between processes related to the disease. Figure 2 shows a low resolution summary (*k *= 10) of the AD network. It indicates that the AD network is represented by an interconnection of several key processes, include protein phosphorylation *(B7)*, cell-cell signaling *(B2, B3)*, and microtubule-based transport and localization *(B1, B5) *processes.

Figure 1 depicts a high resolution functional summary for *k *= 30. Defective transport mechanism has major implications in AD. Consequently, several transport and cytoskeleton organization related cellular processes are represented in the summary *(A11, A22, A24, A26)*. Disrupted transport mechanism affects, among others, synapse organization and vesicle trafficking *(A6, A8, A23)*. In the literature, several lines of evidence explain disruption of transport and its related processes in AD. Amyloid-*β *plaques may lead to hyperphosphorylation of tau proteins, subsequently causing microtubule defects and axonal transport impairment [23]. More strikingly, recent findings indicate that vesicle transport itself play a causative role in pathogenesis of the disease [24]. Glucose metabolic processes *(A20) *is closely linked to microtubule-based processes *(A22, A24)*. The link between bioenergetics and transport in AD has been discussed in [25].

At the center of the summary lies protein folding and calcium ion homeostasis pathways *(A15, A17)*. Protein misfolding is central to AD pathogenesis [26]. Misfolded amyloid-*β *accumulation is shown to induce calcium overload, leading to a variety of structural and functional disruption in neurons [27]. The two functional clusters are among the nodes with the highest degree in the summary. Cell fate processes that trigger or inhibit differentiation and cell fate *(A9, A10, A12) *are also linked to AD [28]. It has been suggested that Wnt signaling dysregulation, a key developmental pathway, leads to reduced synaptic plasticity and function in AD [29]. Processes such as peptide cross-linking and negative regulation of angiogenesis *(A3, A4) *imply vascular roles in AD pathogenesis [30].

From signaling regulation perspective, five major signaling pathways are implicated - small GTPase* (A28)*, Notch *(A14)*, Wnt receptor *(A18)*, glutamate* (A21)*, and G-protein coupled receptor signaling path-ways *(A16)*. Several functional clusters connect with multiple signaling pathways, indicating that signaling pathways crosstalk in AD pathogenesis. For instance, the serine/threonine kinase GSK-3*β*, a potential therapeutic target, is known to be regulator of both the G-protein coupled receptor pathway and the Wnt/*β*-catenin signaling pathway [31]. PS1 may be involved in regulating both Notch and Wnt pathways in AD [32].

The tight interplay of multiple pathways and processes in the aforementioned functional summary of AD network highlights the complexity of the disease. The disease remains poorly understood despite decades of research. While the summary does not suggest causal relationships, in part because of the undirected nature of the FSG, we hope that by having a global, big picture view of process-process interactions, researchers can better identify the causative mechanisms of AD. Most studies considered an aspect of the processes in isolation. An integrative study, however, may lead to a more consistent view of the disease that addresses distinct, often competing hypotheses.

### Inferring functional cluster hubs

Structural information provided by the summaries presents an opportunity to study the topology and connectivity of higher order abstractions of the underlying PPI. Here we analyzed the association patterns of functional clusters in summaries of the global *H. sapiens *PPI. To this end, we generated cellular component (CC) and biological process (BP) summaries of the human network. For each summary type, we varied the level of detail by setting *k *from 50 to 400.

**Figure 10:**
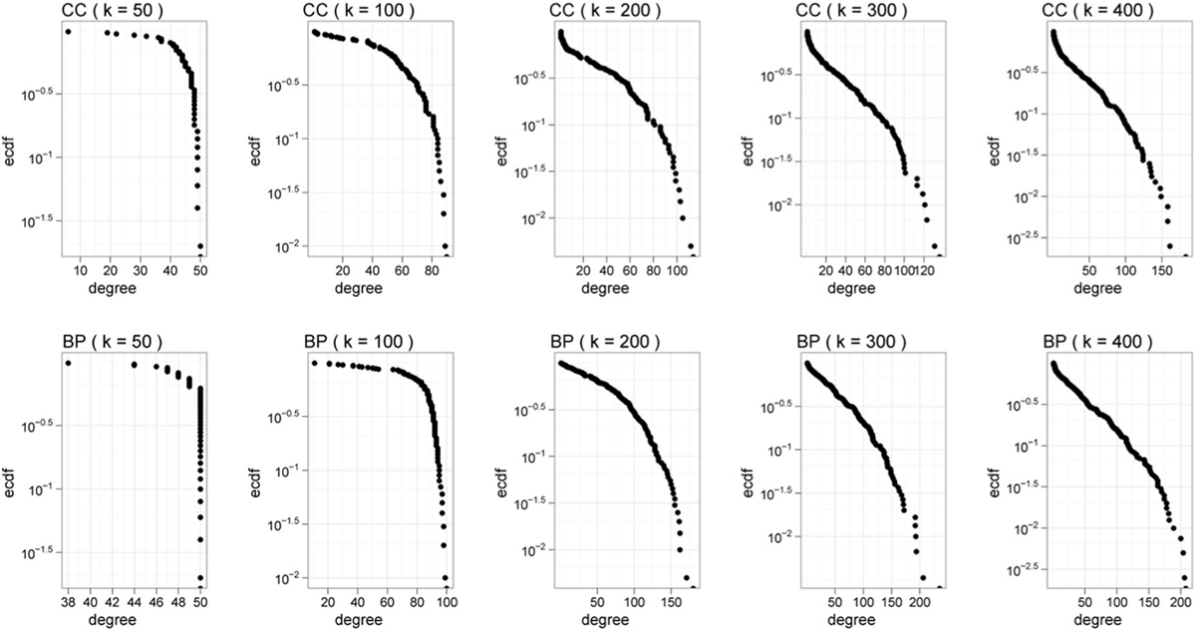
**Connectivity of functional clusters in H. sapiens network.** Functional cluster degree CDF plots for BP and CC summaries at varying cluster granularity. Plots are on a semi-log scale.

Figure 10 shows the frequency-degree plots of the functional clusters at different *k *values. At the broadest level of abstraction (*k *= 50), long-tailed degree distribution of functional clusters is not observed. As level of detail increases to *k *= 400, the smaller and more specific clusters exhibit increasingly pronounced long-tailed distribution characteristics. We note that the CDF plots on a semi-log scale form straight lines at higher *k *values (*k *= 200 and *k *= 400), implying exponential distribution of the cluster degrees.

In light of heavy-tailed distribution of functional cluster degrees at higher *k *values, we identified *functional cluster hubs *in the summary of the human network (*k *= 400) (analogous to identification of protein hubs). While Patil and Nakamura defined hub as proteins having degree of more than 6 [33], we chose a higher threshold such that they correspond to the 15 most connected functional clusters. The list of functional hubs is shown in Table 2.

**Table 2:** High-degree CC and BP functional clusters in the H. sapiens summary (*k *= 400)

CC functional cluster	Degree	BP functional cluster	Degree
Heterogeneous nuclear ribonucleoprotein complex	183	Actin filament bundle assembly	208
Cytosolic large ribosomal subunit	161	Regulation of defense response to virus by virus	206
Cytosolic small ribosomal subunit	158	Negative regulation of catabolic process	204
Coated pit	158	Peptidyl-threonine phosphorylation	200
Mitochondrial nucleoid	149	Signal complex assembly	189
Chaperonin-containing T-complex	148	Positive regulation of protein complex assembly	182
CRD-mediated mRNA stability complex	141	Regulation of nitric oxide biosynthetic process	181
NuA4 histone acetyltransferase complex	136	Glial cell development	178
Actin filament	135	Cell killing	178
Actomyosin	134	Regulation of cytokine-mediated signaling pathway	174
Clathrin coat of coated pit	133	Protein stabilization	174
Nonhomologous end joining complex	124	Actin filament capping	170
Endocytic vesicle membrane	124	Activation of MAPKK activity	169
Nucleosome	124	T cell receptor signaling pathway	164
Nuclear inner membrane	123	Regulation of RNA splicing	164

We observed that CC cluster hubs in *S. cerevisiae *can be categorized into several major functional groups. A significant percentage of the cluster hubs - such as *cytosolic large ribosomal subunit, cytosolic small ribosomal subunit, eukaryotic translation initiation factor 4F complex, preribosome, small subunit precursor, preribosome, large subunit precursor*, and *polysome*- are core to regulation and functioning of protein translation. It is unsurprising that these functional clusters have high degree, since every protein must be translated or regulated by these machinery. The complexity of this mechanism also suggests that it requires many processes to regulate it.

Complexes involved in chromatin remodeling and transcription, including *nuclear nucleosome, Ino80 complex, replication fork protection complex*, astra *complex*, and *Swr1 complex*, are also highly represented. The functional cluster *vacuolar proton-transporting V-type ATPase complex *is known to have diverse roles and is associated with a wide array of processes [34].

Apart from that, we also observed the existence of several 'currency structures', i.e., structures that may be acted upon by proteins from multiple processes. They are generally not specific to a single bio-logical process. We classify clusters *nuclear nucleosome, nuclear microtubule, cytoplasmic microtubule*, and *extracellular region *as such.

Next, we analyzed the bp functional cluster hubs. From Table 2, we found many translation related processes (*regulation of translational initiation, translational elongation, translational termination, tRNA aminoacylation for protein translation, negative regulation of translation, positive regulation of translation, ribosomal small subunit assembly, ribosomal large subunit assembly*). Chromatin assembly and remodeling processes (*nucleosome assembly *and *nucleosome disassembly*) also served as key process hubs. Finally, we found major post-translation protein modification and transport processes, such as *protein refolding*, ATP *synthesis coupled proton transport, cotranslational protein targeting to membrane*, and *proteasome assembly*, acting as hubs.

## Conclusions

In this paper, we propose FUSE, a novel data-driven and generic algorithm for generating functional sum-maries at multiple resolutions from a PPI, providing a high level view of its functional landscape. It exploits mdl principle [14] to generate the "best" summary from both interaction and annotation data by maximizing information gain for a specific resolution. Our experimental study with real-world PPIS revealed that FUSE is effective and have higher accuracy compared to graph clustering techniques in PPI summarization. It is also robust against incomplete interaction knowledge (e.g., AD network in *IntAct*). We note that the graph clustering techniques have the ability to uncover novel complexes, whereas FUSE is designed to determine process-process, complex-complex, and process-complex associations with higher confidence. In this aspect, graph clustering and FUSE play complementary roles. As part of future work, we intend to use FUSE-generated summaries as training data for network comparison of various protein interaction networks at functional level. We believe such comparison may yield interesting findings on function-function and process-process relationships among different networks.

## Methods

### Functional summarization problem

In this section, we formally introduce the functional summarization problem. We begin by defining some terminology that we shall be using in the sequel.

A protein interaction network (PPI) *G *= (*V*, *E*) contains a set of vertices *V *, representing proteins, and a set of edges *E*, representing interactions. An edge has a positive real weight *ω *that represents its interaction strength. Given a GO directed acyclic graph (DAG), denoted as *D*, the ordered set Δ = 〈*a*_1_, *a*_2_, ..., *a_n_*〉 is a topological sort of *D*, where *a_i _*represents a single GO term. The *term association vector *of *v *∈ *V *, denoted by Δ*_v_*, is defined as Δ*_v _*= 〈*a*_1_(*v*), *a*_2_(*v*), ..., *a_n_*(*v*)〉, *a_i_*(*v*) ∈ {0, 1}, such that *a_i_*(*v*) = 1 if and only if the term *a_i _*or its descendants are associated with protein *v*. Otherwise, *a_i_*(*v*) = 0. Note that Δ*_v _*indicates GO terms that are associated with *v*.

### Functional summary of PPI

Given a PPI*G*(*V*, *E*), a *functional summary graph *(FSG) is an undirected graph Θ*_G_*(*S*, *F *) that models the set of higher-order *functional clusters S *and their interactions *F *that underlie the PPI. A *functional cluster *is a subgraph of *G *that shares a particular function/role based on the structure and attribute properties of the subgraph and its constituent proteins. Functional clusters may include complexes, processes, and signaling pathways. A pair of functional clusters may be connected by a web of protein interactions. If the number of interactions are significantly large, then we say that the pair of clusters are *associated*. An FSG Θ*_G _*thus captures higher order modules that comprise the ppi and their interconnections. We now define these concepts formally.

**Definition 1 (Functional Cluster) ***Let V *(*a_i_*) **⊆ ***V denote the set of vertices in G such that v *∈ *V *(*a_i_*) *if and only if *Δ*_v_*[*a_i_*(*v*)] = 1*. The *functional cluster *of a_i _∈ *Δ, *denoted by C*(*a_i_*) ⊆ *G*, *is the subgraph of G that is induced by V *(*a_i_*).

Note that *V *(*a_i_*) represents the set of vertices of *G *that are associated with term *a_i_*∈ Δ. In this paper, we treat *C*(*a_i_*) as a vertex as well. We may also call a functional cluster a *functional subgraph *when we wish to emphasize the fact that it is a graph. Figure 3(b) shows a subset of the possible functional clusters of the PPI in Figure 3(a). Every node in a cluster must share a particular function or attribute. For instance, nodes in functional cluster cytosol share the cytosol term.

**Definition 2 (Functional Summary Graph (FSG)) ***A *functional summary graph *of the underlying protein interaction network G*(*V*, *E*), Θ*_G_, is defined as *Θ*_G _*= (*S, F, P_i_, α*)*, where S is a set of functional clusters and F is a set of edges that links the functional clusters. Let oc_uv _be the number of interactions connecting proteins in C*(*u*) *and C*(*v*)*. Let P_i _be the probability density function of observing o_uv _or more number of interactions between C*(*u*) *and C*(*v*)*. Let β be a significance cut-o parameter (user-defined). Then*, (*C*(*u*), *C*(*v*)) ∈ *F if and only if P_i_*(*X > oc_uv_*) *≤ *2*β/|S|*^2^*. The bijection α *: 1, 2, ..., *m ↔ S is an ordering of S*.

Observe that the aforementioned definition of functional summary includes additional constructs and rules for determining whether two functional clusters are associated. We elaborate on this further. Given a PPI*G*(*V*, *E*), the expected probability of observing an interaction between two randomly drawn protein pair is given by $p_{i}=\frac{2\left|E\right|}{\left|V\right|\left(\left|V\right|-1\right)}$. Let (*C*(*u*), *C*(*v*)) be a functional cluster pair such that members of both clusters were randomly drawn from *V*. If proteins *v*_1 _and *v*_2 _are randomly drawn from *C*(*u*) and *C*(*v*), respectively, then the expected probability of observing a positive interaction between them would also be *p_i_*. Let *n *= *|C*(*u*)*||C*(*v*)*|*. Based on the independent and identically distributed variable (*iid*) assumption, we model the probability of observing *oc *(the number of interactions between *C*(*u*) and *C*(*v*)) as the probability of observing *oc *positive interactions after *n iid *trials, representing *n *pairwise interaction trials between proteins in *C*(*u*) and *C*(*v*). Hence, the probability of *oc *or more positive interactions between *C*(*u*) and *C*(*v*) can be modeled using a binomial distribution:

$$P_{i}\left(X>oc_{uv}\right)=\overset{n}{\underset{i=oc_{uv}}{\sum }}\left(\begin{array}{c} n\\ i \end{array}\right){p_{i}}^{i}{\left(1-p_{i}\right)}^{n-i}$$

This *p - value *is used to assess the *association significance *between a pair of functional clusters. Given a set containing *k *clusters, association significance between $\frac{1}{2}k\left(k-1\right)$ pairs of clusters would have to be tested. To this end, we applied Bonferroni correction to account for multiple testing. Given the *significance cut-off β*, a pair of functional clusters is *significantly associated *if

$$P_{i}\left(X>oc\right)\;\leq \;2\beta /k\left(k-1\right)\approx 2\beta /k^{2}$$

Observe that although we have adopted a simple model to assess cluster-cluster association, the aforementioned definition is general enough to encompass more sophisticated association models.

**Example 1 **Figure 3(d) shows an FSG consisting 5 functional clusters. Any edge between two functional clusters exists when *P_i_*(*X > oc_uv_*) ≤ 2*β *=|*S*|^2^, implying that more edges connect proteins between the functional clusters than expected in random.

### Problem statement

The functional summarization problem is the problem of finding Θ*_G _*that best represents the underlying PPI subject to a *summary complexity constraint*. To model this problem, we propose a profit maximization model that aims to find Θ*_G _*= (*S*, *F*, *P_i_*, *α*) by maximizing information profit under a budget constraint. Every protein *i *∈ *V *is assigned a non-negative *information budget b*, which represents the information it contains. Let *S*_Δ _be the set of functional clusters induced from Δ. Every functional cluster *C*(*u*) ∈ *S*_Δ _is assigned a non-negative *structural information value ψ*^*C*(*u*)^(to be defined later), which represents the amount of structural information contained within the functional subgraph. When a functional cluster *C*(*u*) is added to the summary, for every protein *i *∈ *V *(*u*), a portion of *b *is taken out and added to summary information gain. This represents new information added to the summary. The amount to take depends on ψ^*C*(*u*)^. Imposing information budget *b *limits the amount of information a protein can provide. A parameter 0 ≤ *d *≤ 10 is also introduced to penalize redundancy. By doing so, repeated representation of a protein *i *yields reduced information gain, modeling diminishing returns. Based on this profit model, we construct the set of functional clusters that maximizes profit while satisfying the constraints.

**Definition 3 (Functional Summarization Problem) ***Let K_i _be a set of functional clusters such that C*(*u*) ∈ *K*_*i *_*if and only if i*∈ *C*(*u*). *For every C*(*u*) ∈ *S*_Δ_, *let ψ*^*C*(*u*) ^*be the structural information value of C*(*u*)*. Given a protein interaction network G*(*V*, *E*) *and user-defined parameters b, d and k, the functional summarization problem constructs a k-cluster *FSG Θ*_G _*= (*S*, *F*, *P_i_*, α) *that satisfies the following optimization problem:*

(2) $$\begin{array}{c} maximize\underset{i\in V}{\sum }\overset{\left|S\right|}{\underset{j=1}{\sum }}p\left(i,j\right)\\ where\\ b\left(i,m\right)=\left\{\begin{array}{cc} \frac{d}{10}\left(b\left(i,m-1\right)-p\left(i,m-1\right)\right) & \begin{array}{c} if\;m>1,\\ \alpha_{S}\left(m-1\right)\in K_{i} \end{array}\\ b\left(i,m-1\right) & \begin{array}{c} if\;m>1,\\ \;\alpha_{S}\left(m-1\right)\notin K_{i} \end{array}\\ b & if\;m=1 \end{array}\right)\\ and\\ p\left(i,m\right)=\left\{\begin{array}{c} \psi^{\alpha_{\text{S}}\left(m\right)}\\ b\left(i,\;m\right)\\ 0 \end{array}\;\begin{array}{c} if\;b\left(i,m\right)\geq \psi^{\alpha_{\text{S}}\left(m\right)}and\;\alpha s\left(m\right)\in K_{i}\\ if\;b\left(i,m\right)<\psi^{\alpha_{\text{S}}\left(m\right)}and\;\alpha_{S}\left(m\right)\in K_{i}\\ \alpha_{S}\left(m\right)\notin K_{i} \end{array}\right)\\ subject\;to\\ |S|\;=k\\ S\subset S_{\Delta } \end{array}$$

We elaborate on how the *structural information value ψ*^*C*(*u*) ^is assigned. A functional cluster *C*(*u*) and its protein constituents share a common function *u*, and thus vertices in the subgraph are considered homogeneous attribute wise. However, it does not imply that the functional subgraph is structurally cohesive (dense). Proteins having common function *u *may still be weakly interacting. This may be due to the fact that *u *itself may indicate a general function (e.g., 'protein binding') which is a common attribute to a large number of proteins that do not interact with each other. We argue that structurally cohesive functional clusters contain more information than those which are loosely interconnected. The argument for this is based on the MDL principle, whereby clusters that have higher than expected cohesiveness will have higher information content because of the lower probability of observing a random cluster having the same cohesiveness. However, we make the following exception - a functional cluster with lower than expected cohesiveness is not deemed structurally informative.

Since the optimization problem must choose among a set of functional clusters, we are not concerned about the actual p-value of observing a subgraph having such interaction density. Instead, we only need a measure that allows us to compute the relative ranking of the functional clusters by their information content. Such simplification leads to much greater computation efficiency. We define the *structural information value *of a functional cluster *C*(*u*) as follows.

**Definition 4 (Structural Information Value) ***Let ω_ij _be the edge weight of *(*i, j*) ∈ *E*. *The *structural information value *of a functional cluster C*(*u*)*, denoted by ψ*^*C*(*u*)^, *as ψ*^*C*(*u*) ^= *p*^*C*(*u*) ^*where*

$$\rho^{C\left(u\right)}=\frac{\sum_{i,j\in C\left(u\right)}\omega_{ij}}{\left|C\left(u\right)\right|}$$

**Algorithm 1 **Algorithm FUSE

**Input: ***G*, Δ, *D*, *k*, *b*, *d*, *β*

**Output: **Θ*_min_*

1: Let *S *= empty set

2: Let *B*_map _= set of pairs (*i*, *b*) for each *i *∈ *V*

3: Assign *ψ*^*C*(*u*) ^and *c*^*C*(*u*) ^for each *C*(*u*) ∈ *S*_Δ_

4: *i *= 0

5: **while ***i < k ***do**

6:    (*C*_min_, *B*_map_) = **MapProfit**(*S*_Δ_, *B*_map_, *d*, *|V|*, *k *)

7:    Remove *C*_min _from *S*_Δ_

8:    Add *C*_min _to *S*

9:    *i *= *i *+ 1

10: **end while**

11: **for ***C*(*i*), *C*(*j*) ∈ *S ***do**

12:    **if ***C*(*i*) ≠ *C*(*j*) and *P*_*i*_(*X > oc*_*C*(*i*)*C*(*j*)_) *≤ *2*β = |S|*^2 ^**then**

13:       Add edge (*C*(*i*), *C*(*j*)) to *F*

14:    **end if**

15: **end for**

^*ρC*(*u*) ^is the *ratio association *[35] score of *C*(*u*), a standard graph clustering objective we adopt that indicates the structural density of *C*(*u*). At first glance, it may seem that the structural information value should be defined as *ψ*^*C*(*u*) ^= *ρ*^*C*(*u*) ^*- ρ*^random^, where *ρ*^random ^is the *expected structural density *of a random cluster. However, we ignore *ρ*^random ^for the following reason. In scale-free and Erdős-Rényi graphs, the self-information *- *log *P *(*ψ*^*C*(*u*)^) is a positive non-decreasing function of *ψ*^*C*(*u*) ^for *ψ*^*C*(*u*) ^*>*0. Hence, *ψ*^*C*(*u*) ^can be used to compare the self-information between two functional clusters without having to determine the probability density function of the interaction distribution of a subgraph. Given *a_i_*, *a_j _*∈ Δ, *C*(*a_i_*) is deemed more informative than *C*(*a_j_*) if and only if *ψ*^*C*(*a*_*j*_) ^*> ψ*^*C*(*a*_*i*_) ^and *ψC*(*a_j _*) *>*0. If both *ψ*^*C*(*aj*) ^and *ψ*^*C*(*ai*) ^are negative, it does not matter whether one is more informative than the other, since both have structural density less than that of random networks. As such, for symmetry, we also deem that *C*(*a*_*i*_) is *more informative *than *C*(*a*_*j*_) if and only if *ψ*^*C*(*a*_*j*_) ^*> ψ*^*C*(*a*_*i*_) ^for *ψ*^*C*(*aj*) ^*≤ *0. Therefore, when comparing the structural density between two clusters, *ρ*^random ^can be omitted from *ψ*^*C*(*u*) ^and *ψ*^*C*(*u*) ^is simply *ρ*^*C*(*u*)^.

**Example 2 **Suppose we wish to summarize the PPI in Figure 3(a) into a 3-node summary (*k *= 3). If clusters apoptosis, receptors, and TGF-beta are chosen--instead of the clusters in Figure 3(c)--we can see that the profit obtained is suboptimal. Information budget for proteins b, c are depleted due to redundancy, while information budget for proteins d, e, g, i are untapped. In contrast, functional summary in Figure 3(c) is relatively more optimal, as not only the set of clusters maximizes profit through superior coverage and minimal redundancy, but it also maximizes profit through higher structural information (e.g., the cluster transcription is structurally dense compared to apoptosis).

**Algorithm 2 **The *Map Profit *procedure.

**Input: ***S*_Δ_, *B*_map_, *d*, *|V |*, *k*

**Output: ***C*_min_, *B*_map_

1: Let *p*_max _= 0

2: **for ***C*(*u*) ∈ *S*_Δ _**do**

3:    Let *B*_temp _= *B*_map_

4:    Let *p *= 0

5:    **for ***i *∈ *V *(*u*) **do**

6:       Let (*i*, *b*(*i*)) ∈ *B*_temp _and *p*(*i*) = *b*(*i*) *- ψ*^*C*(*u*)^

7:       **if ***p*(*i*) *>*0 **then**

8:          *p *= *p *+ *ψ*^*C*(*u*)^

9:          *b*(*i*) = *b*(*i*) *- ψ*^*C*(*u*)^

10:       **else**

11:          *p *= *p *+ *b*(*i*)

12:          *b*(*i*) = 0

13:       **end if**

14:    **end for**

15:    $c^{C\left(u\right)}={\left(\left|V\left(u\right)\right|-\frac{\left|V\right|}{k}\right)}^{2}$

16:    *p *= *p *-*c*^*C*(*u*)^

17:    **if ***p*_max _*< p ***then**

18:       *p*_max _= *p*

19:       *C*_min _= *C*(*u*)

20:    **end if**

21: **end for**

22: for *i *∈ *V*_min _**do**

23:    Let (*i*, *b*(*i*)) *B*_map _and *p*(*i*) = (*d*/10)(*b*(*i*) - *ψ*^*C*(*u*)^)

24:    **if ***p*(*i*) *>*0 **then**

25:       *b*(*i*) = (*d*/10)(*b*(*i*) *-ψ*^*C*(*u*)^)

26:    **else**

27:       *b*(*i*) = 0

28:    **end if**

29: **end for**

30: **return **(*C*_min_, *B*_map_)

### The algorithm FUSE

The profit maximization problem is a variation of the *budgeted maximum coverage problem *[36], which is an np-hard problem. To permit a tractable solution, let us first consider a straightforward greedy approach. The initial FSG is an empty graph. Given the input protein interaction network *G*, *ψ*^*C*(*u*) ^for each functional cluster *C*(*u*) ∈ *S*_Δ _are computed. The algorithm then iteratively selects the functional cluster that leads to greatest increase in net profit of the summary. Each time a functional cluster *C*(*u*) is selected, the FSG and budget information *b*(*i*) for every protein *i *∈ *V *(*u*) is updated. Once *k *clusters has been selected, the algorithm terminates by generating the FSG.

A major weakness of the aforementioned approach is that it tends to be "overenthusiastic" in selection of functional clusters during early iterations. Functional clusters that are too large or too small may be selected at early iterations resulting in very poor cluster choices at later iterations due to limited information budget and summary size (*k*) constraint. Hence, our proposed algorithm adds a *complexity cost *to each chosen cluster. Given graph size *|V | *and summary size *k*, the *expected cardinality *of a functional cluster in the summary is defined by $E\left[\left|C\right|\right]=\frac{\left|V\right|}{k}$. Then the *size deviation cost*, denoted as *c*^*C*(*u*)^, is defined as the square of the deviation of |*C*(*u*)| from *E*[|*C*|]. That is, $c^{C\left(u\right)}={\left(|V\left(u\right)|-\frac{\left|V\right|}{k}\right)}^{2}$. Observe that the greater the difference between |*V *(*u*)| and *E*[|*C*|], the less likely it is to be part of a summary of *k*-granularity. Clusters whose size deviates too much from the expected cardinality are penalized and therefore less likely to be selected. This reduces the chance of having too less or too much information budget remaining during the later iterations of the greedy heuristic.

The aforementioned intuition is realized in FUSE as outlined in Algorithm 1. It consists of three phases, namely, the *initialization *phase, the *greedy iteration *phase, and the *summary graph construction *phase. In the initialization phase (Lines 1-3), *ψ*^*C*(*u*) ^and *c*^*C*(*u*) ^for each functional cluster *C*(*u*) *S*_Δ _are computed. The greedy iteration phase (Lines 4-10) involves iterative addition of functional clusters into *S *in a greedy manner as described above. The best candidate functional cluster for the current round (*C*_min_) is determined through the subroutine **MapProfit **(Line 6). This step also maintains the information profit of *S *and the remaining information budget of every *v *in *G *through a persistent *pro t map *(*B*_map_). *C*_min _is then removed from the candidate pool *S*_Δ _and added to the solution set *S *(Lines 7-8). Finally, the summary graph construction phase (Lines 11-15) computes *F *to generate the FSG Θ_min_.

The **MapProfit **procedure is outlined in Algorithm 2. In order to identify the best candidate cluster of the current iteration round, it evaluates every cluster in the candidate pool by evaluating its profit gain potential (Lines 1-21). First, the amount of information to extract from each protein's information budget pool (*b*(*i*)) is computed (Lines 7-13). Next, the potential profit gain is adjusted to compensate for the complexity cost (Lines 15-16). After *C*_min _is found, the profit map is recomputed to commit the changes made to the information budget map due to the selection of *C*_min _(Lines 21-29).

**Theorem 1 ***Algorithm *FUSE*takes O*(|*S*_Δ_|^2^|*V *|^2^) *time in the worst case*.

### Proof of theorem 1

In the initialization phase, *ψ*^*C*(*u*) ^for each *C*(*u*) *S*_Δ _has to be computed. Each *C*(*u*) may contain up to |*E*| edges and |*V *| vertices. In Algorithm 1, *ψ*^*C*(*u*) ^for each *C*(*u*) *S*_Δ _takes *O*(|*E*|) time. Thus, thus the total complexity for this procedure is *O*(|*E*||*S*_Δ_| + |*V *||*S*_Δ_|) time.

In the greedy iteration phase, the **MapProfit **subroutine defined in Algorithm 2 is evaluted *k *times. In Algorithm 2, lines 2-21 require *O*(|*S*_Δ_||*V *|). Lines 22-29 require *O*(|*V *|) time. Thus, Algorithm 2 takes *O*(|*S*_Δ_||*V *| + |*V *|) time. The iteration phase, as such, takes *O*(*k*|*S*_Δ_||*V *| + *k*|*V *|) time in total.

Finally, the summary graph construction phase involves pairwise significance evaluation of the resultant functional cluster set. This involves evaluation of all edges between *k*-pairwise functional clusters of the summary. Each significance *P_i_*(*X > oc_uv_*) test requires a single-pass evaluation of edges connecting a pair of clusters. At worst case, this takes *O*(*|E|*) time. The summary graph construction phase therefore require *O*(*k*^2^|*E*|) time.

The FUSE algorithm, as whole, takes *O*(|*E*||*S*_Δ_| + |*V *||*S*_Δ_*| *+ *k|S*_Δ_*||V | *+ *k|V | *+ *k*^2^*|E|*) time. In the worst case scenario of *|E| *= *|V |*^2 ^and *k *= *|V |*, the algorithm takes *O*(*|S*_Δ_*||V | *+ *|S*_Δ_*||V |*^2 ^+ *|V |*^2 ^+ *|V |*^4^) time, implying a polynomial time complexity at worst possible case.

### Evaluation metrics

We used the *coverage *metric to evaluate the fraction of the annotated protein interaction network covered by a summary. A functional summary with high coverage is desirable because it is more representative of the underlying interaction network than a summary with low coverage. The coverage of a functional summary Θ is defined as:

(3) $$coverage\left(\Theta \right)=\frac{\left|{⋃}_{C\left(u\right)\in S_{\Theta }}V\left(u\right)\right|}{\left|{⋃}_{C\left(u\right)\in S_{\Delta }}V\left(u\right)\right|}$$

The coverage is the ratio of the total number annotated proteins in the summary over the total number of annotated proteins in the protein interaction network.

The *redundancy *metric is the average number of functional clusters each protein belongs to. This is an indicator of the amount of cluster overlap in the summary. Redundancy of Θ is defined as:

(4) $$redundancy\left(\Theta \right)=\frac{\underset{C\left(u\right)\in S_{\Theta }}{\sum }\left|V\left(u\right)\right|}{\left|{⋃}_{C\left(u\right)\in S_{\Theta }}V\left(u\right)\right|}$$

A summary Θ with no overlapping clusters will have lowest possible redundancy value of 1, where every protein is assigned to exactly one cluster. A summary with high redundancy is undesirable, because a summary with many highly overlapping clusters is less intuitive and more complicated.

The following well-known evaluation metrics are also used - *precision and recall*. These are well known statistical measures to indicate accuracy and completeness. Precision, a measure of exactness, is defined as $precision=\frac{truepositive}{truepositive+falsepositive}$. Recall, a measure of completeness, is defined as $recall=\frac{truepositive}{truepositive+falsenegative}$. If a cluster *C*(*i*) is assigned with the function *i*, then any protein *p *∈ *C*(*i*) that is not annotated with *i *or its descendants is deemed a false positive. If *p *∈ *C*(*i*) is annotated with *i *or descendants, it is a true positive. Likewise, a protein *p *∈ *V *that is annotated with *i *but not in *C*(*i*) is deemed a false negative. Here, proteins without annotations are not taken into consideration.

## Competing interests

The authors declare that they have no competing interests.

## Authors' contributions

BSS and SSB conceived of the study. BSS designed the algorithm and performed data analysis. SSB, HY and CFD provided critical input and made revisions to the study and manuscript. BSS and SSB wrote the manuscript. All authors read and approved the final manuscript.
